# Clinical management, expectations, and satisfaction of patients with moderate to severe allergic rhinoconjunctivitis treated with SQ-standardized grass-allergen tablet under routine clinical practice conditions in Spain

**DOI:** 10.1186/s12948-016-0057-9

**Published:** 2017-01-06

**Authors:** Tomás Chivato, Pedro Álvarez-Calderón, Carmen Panizo, Ricardo Abengozar, César Alías, Ali Al-Baech, José Arias-Irigoyen, M. José Caballero, Lluis Conill, Silvia de Miguel, Rafael Laguna, Joan Martínez-Benazet, Francisco Matoses, Jose Camilo Martínez-Alonso, Lourdes Mendizábal, Celsa Pérez-Carral, Carlos Puerto, Joan Serra-Batllés, Adolfo Vélez, Jonathan Vicente, Fernando de la Torre

**Affiliations:** 1 Decanato. Facultad de Medicina, Facultad de Medicina Universidad CEU San Pablo. Grupo Hospitales HM, Avda. Montepríncipe s/n, Boadilla del Monte, 28668 Madrid, Spain; 2 Hospital Clínico Universitario de Santiago, La Coruña, Spain; 3 Hospital Ntra Sra del Prado, Toledo, Spain; 4 Hospital Virgen del Valle, Toledo, Spain; 5 Clínica Corachán, Barcelona, Spain; 6 Private Office, Huelva, Spain; 7 Clínica ASISA, Córdoba, Spain; 8 Hospital Can Misses, Ibiza, Spain; 9 Policlínico Salud 4, Madrid, Spain; 10 Private Office, Madrid, Spain; 11 Hospital Ntra Sra de Meritxell, Escaldes-Engordany, Andorra; 12 Hospital General, Valencia, Spain; 13 Hospital Virgen de la Concha, Zamora, Spain; 14 Clínica Virgen de Fátima, Seville, Spain; 15 Hospital Comarcal da Costa, Lugo, Spain; 16 Private Office, Asturias, Spain; 17 Hospital de Vic, Barcelona, Spain; 18 Hospital San Juan de Dios, León, Spain; 19 Hospital General, Soria, Spain; 20 ALK-Abelló, S.A, Madrid, Spain

**Keywords:** Allergic rhinitis, Allergen immunotherapy tablet, Satisfaction, Expectations, Clinical management

## Abstract

**Background:**

Sublingual immunotherapy has been proven as a well-tolerated and effective treatment for allergic rhinitis. Within this type of treatment, GRAZAX^®^ is the most documented product in terms of safety and efficacy. The objective of this study was to identify the patients’ expectations and level of treatment satisfaction, as well as the clinical management of patients with moderate/severe allergic rhinoconjunctivitis treated with GRAZAX^®^.

**Methods:**

This was a non-interventional, observational, multi-centre, open-label study involving a total of 131 adult patients aged 18–66 years with confirmed diagnosis of grass-allergy and initiated treatment with GRAZAX^®^ between June 2010 and April 2011.

**Results:**

In the pollen season after starting treatment, 56.6% of patients stated that their symptoms were much less/less intense, 86% needed less symptomatic medication for control of their symptoms, and 74.4% manifested to have improved (quite/a lot) as regards their allergic disease since treatment was initiated as compared with previous grass pollen season. The patient satisfaction with GRAZAX^®^ was measured using a visual analogue scale (VAS) between 0 (minimum satisfaction) and 100 (maximum satisfaction) comprising five different items: effectiveness, tolerability, cost, convenience and overall satisfaction. The results obtained for each item were [mean (SD)]: 74.7 (18.1), 70.3 (36.1), 39.3 (25.8), 86.2 (12.6), 78.4 (15.8) respectively. The patient’s level of satisfaction is highly influenced, especially in terms of assessment of effectiveness, tolerability and convenience, by the information provided by the specialist.

**Conclusions:**

In summary, it can be concluded that improved communication leads to increased patient knowledge, greater patient compliance, and increased patient satisfaction.

## Background

Despite not being considered a life-threatening disease, allergic rhinitis or rhinoconjunctivitis cause functional problems (physical, emotional, social, and occupational) that tend to worsen with time. Moreover, this disease represents a high cost burden for healthcare systems, often exceeding those derived from more serious conditions; allergic rhinoconjunctivitis is very common [1], affecting around one in four people of the total population. In a large-scale study that included over 9500 patients from Western European countries, the prevalence of allergic rhinitis was found to be around 23%. Because it is frequently considered not to be a life-threatening disease, it often remains undiagnosed, resulting in inadequate control of the symptoms [1].

From an economic point of view, the consequences of allergic rhinoconjunctivitis are considerable in terms of direct (treatments and visits to the GP/specialist) and indirect (i.e., sick leave from work or school, sleep disorders, and disruption of daily activities) costs.

Grasses are one of the most common causes of allergic rhinitis, having a clear impact on patients’ quality of life [2]. There are various options for the management of this condition: symptomatic medication for the relief of the symptoms and avoidance of allergen exposure. On the other hand, the underlying cause of the allergic disease can be targeted through allergen-specific immunotherapy. Although proven effective, subcutaneous administration of the allergen can be uncomfortable and time-consuming. Sublingual allergen immunotherapy has been the subject of clinical development over the last 20 years. A Cochrane review published in 2011 [3] concluded that the use of sublingual immunotherapy for allergic rhinitis and rhinoconjunctivitis is effective and is not associated with significant adverse events.

Regarding sublingual immunotherapy, the grass allergen tablet GRAZAX^®^ (*Phleum pratense* 75,000 SQ-T/2800 BAU, ALK, Denmark) is the most documented product in terms of safety and efficacy in children and adults [4–12].

However, several aspects of this therapeutic alternative, which tend to be closely associated with therapeutic adherence, have not been thoroughly examined, as for example: patients’ expectations and their knowledge concerning a certain treatment [13, 14], the administration routes for immunotherapy [15], patient satisfaction with this treatment [16], and certain clinical management and patient education factors that may help reinforce positive patient behaviour regarding the treatment [17, 18].

The purpose of this study is to determine the expectations and level of satisfaction of patients (using the data collected from a questionnaire), as well as to collect information regarding the clinical management of patients with moderate/severe allergic rhinoconjunctivitis treated with GRAZAX^®^.

## Methods

### Design, treatment, and patients

Non-interventional, observational, multicenter, open-label study that included one hundred and thirty-one (131) adult patients aged between 18 and 66 years. All study subjects had a confirmed diagnosis of grass pollen allergy, reached through routine clinical practice (positive prick test and/or specific IgE) and had initiated treatment with GRAZAX^®^ between June 2010 and April 2011 (i.e., had started treatment at least 2 months before the beginning of the 2011 grass pollen season). Two patients were excluded from the study because they were under 18 years of age.

Written informed consent was obtained from all patients before their inclusion in the study. The corresponding health authorities and the ethics committee approved the study.

Patients enrolled in the study were provided with a questionnaire comprising 18 questions and a visual analogue scale (VAS). The questionnaire was answered by the study subjects and aimed to assess their state of health and knowledge and expectations regarding the treatment with GRAZAX^®^; the VAS scoring ranged from *not at all satisfied* (0) to *maximum satisfaction* (100).

### Variables

In this exploratory, descriptive study, no primary endpoint was established. Instead, a cluster of three relevant variables were assessed using an ad hoc questionnaire

- *Current patient satisfaction* to our knowledge, there is no standardised validated tool for this patient population. Thus, this variable was assessed through items specifically designed for this study. *The* ad hoc items in the study included Likert-type response options or visual analogue scorings.
- *Patients’ expectations at the start of treatment and at present* a series of ad hoc items were drawn up. Data regarding the information patients received about their illness and treatment were also collected.
- *Clinical management received by patients* certain characteristic aspects of patients’ perception regarding their clinical management.

Study data was collected retrospectively the year before the inclusion of the patients in the study. There was no follow-up of the participants as the study consisted of a single visit for data collection. Sociodemographic and clinical data were also collected.

### Statistical analysis

Nonparametric tests (bilateral Kolmogorov–Smirnov test with 95% confidence level) were used to assess the normal distribution of the variables. Statistical inference was obtained by bivariate or multivariate analyses. The bivariate analyses were parametric (Chi squared, Student’s t distribution, ANOVA) or bilateral nonparametric (Fisher’s exact, Mann–Whitney, Kruskal–Wallis), with a 95% confidence level.

## Results

### Characteristics of patients

Mean age was 33.9 ± 12.9 years (53.5% female and 46.5% male). All study patients suffered from allergic rhinitis (see the characteristics of the disease in Table 1), as diagnosed by the ARIA guidelines [2]. Sixty-seven (67) patients had allergic asthma associated with rhinitis (73.1% intermittent, 14.9% mild persistent, 11.9% moderate, as per the Spanish Guidelines for the Management of Asthma -GEMA-) [19]. Median of time of treatment: 13 months (Q1–Q3: 11–17).

**Table 1:** Clinical characteristics of the rhinitis

Characteristics of allergic rhinitis	% of patients
Intermittent	43.7
Persistent	56.3
Mild	20.2
Moderate-severe	79.8
Sleep interference	24.0
Interference with daily activities	43.4
Interference with working activities or studies	42.6
Troublesome symptoms	68.2
Symptomatic medication used for rhinitis	
Topical antihistamines	17.5
Oral antihistamines	73.0
Topical corticosteroids	44.4
Oral corticosteroids	4.8
Antileukotrienes	17.5

35.7% of study patients were monosensitized to grass, from which 64.3% were sensitized to grass and other allergens (these patients were considered as polysensitized). The most frequent allergens affecting the latter group were pollens (*Olea*: 33.7%; *Cupressus*: 26.5%; *Plantago*: 24.1%), mites (*D. pteronyssinus*: 28.9%), moulds (*Alternaria*: 9.6%), and epithelia (cat: 24.1%; dog: 20.5%).

### Patients’ expectations at the beginning of the treatment and at present

Patients were asked about their knowledge and expectations regarding their treatment with GRAZAX^®^. The results are summarized in Table 2.

**Table 2:** Knowledge and expectations regarding the allergen immunotherapy tablet

	n	%
*In your opinion, which is the aim of an allergen treatment?* (n = 126)		
Improve quality-of-life	83	65.9
Reduce the number of attacks	34	27.0
Prevent life-threatening events	5	4.0
Avoid the development of new allergies	4	3.2
*What do you expect from your treatment with the allergen immunotherapy tablet?* (n = 128)		
Complete cure of the allergy	79	61.7
Some improvement of the symptoms	33	25.8
Avoid the development of new allergies	4	3.1
Prevent the onset of asthma symptoms	12	9.4
*When do you think the allergen immunotherapy tablet will start to be effective?* (n = 123)		
In a few days or weeks	41	33.3
In a few months	66	53.7
In a few years	16	13.0
*Do you think the treatment could be dangerous or cause adverse events?* (n = 115)		
The *allergen immunotherapy tablet* is safe	34	29.6
Sometimes	39	33.9
Rarely	42	36.5

When the answers in different subgroups of patients based on age, sex, mono-/polysensitized, years of evolvement of the allergic disease, or type and severity of allergic rhinitis were analysed, statistically significant differences were only found for the expectations regarding treatment. Thus, whereas 41.7% of patients with mild rhinitis expected a complete cure, this percentage increased to 64.9% (*p* = 0.042) in patients with moderate-severe rhinitis.

### Clinical management of the patients

All study patients were asked on the type of information received from their allergologist regarding their allergic disease and treatment with GRAZAX^®^. The main results are included in Table 3.

**Table 3:** Clinical information received by the patients

	n	%
*Did you receive information about your disease before starting the treatment with the allergen immunotherapy tablet?* (n = 128)		
Yes, in detail	114	89.1
Yes, a little	14	10.9
*Did you receive information about the different treatments for your disease?* (n = 129)		
Yes, in detail	102	79.1
Yes, a little	25	19.4
I received no information	2	1.6
*Did you receive information about the characteristics of the allergen immunotherapy tablet?* (n = 128)		
Yes, in detail	107	83.6
Yes, a little	20	15.6
I received no information	1	0.8
*Did you receive written information about your disease or its treatment?* (n = 128)		
Yes	58	45.3
No	70	54.7

During the pollen season, 56.6% of patients stated their symptoms were much less/less intense, 86% required less symptomatic medication for the control of their allergic symptoms, and 74.4% stated the allergic disease had improved (quite/a lot) since the initiation of the treatment with GRAZAX^®^ in comparison with the previous grass pollen season.

### Level of satisfaction of patients treated with GRAZAX^®^

Patients´ satisfaction with GRAZAX^®^ was measured using a visual analogue scale (VAS) with a range of scores between 0 (minimum satisfaction) and 100 (maximum satisfaction) comprising five items: effectiveness, tolerability, cost, convenience, and overall satisfaction. The following results, expressed as means (SD), were obtained for each item: 74.7 (18.1), 70.3 (36.1), 39.3 (25.8), 86.2 (12.6), 78.4 (15.8), respectively. No statistically significant differences were found between these variables regarding the age and sex of the patients.

Considering the duration of the disease (<6 years, 6–12 years, or >12 years), significant differences were found for the variable “effectiveness” (78.2 (16.3) vs. 76.4 (18.3) vs. 69.7 (19.0), respectively; *p* = 0.025). Statistical differences were also found for the degree of severity of the allergic rhinitis (Table 4).

**Table 4:** Degree of satisfaction with the allergen immunotherapy tablet *as per* the severity of the allergic rhinitis (VAS scale: 0 -not at all satisfied-, 100 -maximum satisfaction-)

AR type	n	Effectiveness	Adverse effects	Cost	Convenience	Overall
		Mean (SD)	Mean (SD)	Mean (SD)	Mean (SD)	Mean (SD)
Mild	24	80.42 (16.15)	83.54 (30.16)	55.21 (25.64)	89.17 (13.73)	82.29 (17.00)
Moderate/severe	93	72.42 (18.85)	66.02 (37.18)	36.61 (24.36)	85.86 (12.33)	77.08 (16.17)
*p* value^1^		0.032	0.041	0.002	0.092	0.058

^1^The p value was calculated using bilateral Student’s parametric t test (or the nonparametric Mann–Whitney U test) with a 95% confidence level

Table 5 shows how patients´ satisfaction level is highly influenced by the information provided by the specialist, particularly in terms of assessment of effectiveness and convenience.

**Table 5:** Relationship between patient satisfaction and disease- and treatment-related information received

	n		Effectiveness	Adverse events	Cost	Convenience
		Mean (SD)	Mean (SD)	Mean (SD)	Mean (SD)	Mean (SD)
Before starting the treatment, did your doctor speak to you about the disease?						
Yes, at length	112	76.34 (17.6)	69.91 (36.35)	40.0 (25.86)	86.74 (13.10)	79.63 (15.12)
Yes, a little	14	62.50 (17.84)	77.86 (31.85)	33.2 (25.91)	82.50 (8.03)	72.14 (15.65)
*p* value^1)^		0.002	0.446	0.272	0.050	0.108
Did your doctor speak to you about the different possible treatments for your disease?						
Yes, at length	100	76.70 (17.56)	70.40 (36.57)	40.45 (26.45)	87.70 (12.50)	78.85 (15.73)
Yes, a little	25	69.40 (17.52)	68.80 (36.15)	35.20 (23.07)	80.00 (11.99)	77.72 (16.20)
*p* value^1^		0.012	0.959	0.577	0.007	0.491
Did your doctor speak to you about the characteristics of GRAZAX^®^?						
Yes, at length	105	76.33 (18.00)	68.62 (37.65)	39.38 (26.47)	87.57 (12.39)	78.84 (15.95)
Yes, a little	20	69.50 (15.30)	77.75 (28.07)	39.50 (22.59)	78.75 (12.23)	77.50 (15.09)
*p* value^1^		0.023	0.904	0.460	0.008	0.237

^1^The p value was calculated using the bilateral ANOVA parametric test (or nonparametric Kruskal–Wallis) with a 95% confidence level

Finally, overall patient satisfaction with GRAZAX^®^ with respect to previous immunotherapy treatments showed that 56.36% of the patients considered GRAZAX^®^ as “much better” and 30.9% as “better”.

## Discussion

Allergic rhinoconjunctivitis and rhinitis are independent risk factors for asthma. These two conditions increase the social and economic impact of asthma and represent an important burden for the healthcare system [2, 20]. Data on drug intake, healthcare resources, days of work missed, and loss of productivity associated to allergic rhinitis were collected by the Spanish Society of Allergy and Clinical Immunology (SEAIC).

The management of allergic rhinitis involves different strategies, including patient education, allergen avoidance, and symptomatic and etiological treatments. Within this latter option, GRAZAX^®^ is the only product authorized for treating grass pollen allergy with a registered disease-modifying effect [21]. According to the results obtained in the clinical trials performed with GRAZAX^®^, a significant clinical benefit is achieved after the first 4 months of treatment [9]. For this reason the authors assumed that 1 year of treatment could be an acceptable time for evaluating the satisfaction and expectations of patients.

The healthcare system could benefit from an annual increase in the number of patients treated with specific immunotherapy in terms of additional cost savings and improved health status for the patients [20]. In this study we show that 86% of patients treated with GRAZAX^®^ take less symptomatic medication after starting the treatment, which implies lower costs for the national healthcare system. These findings are in line with a previous study by Donahue et al. [22]. who found immunotherapy to be a less cost-intensive strategy than standard pharmacotherapy over a 10-year period. Treatment with GRAZAX^®^ has been described in the literature to be a cost-effective intervention for the prevention of grass pollen-induced rhinoconjunctivitis [23, 24]. However, factors related to patient satisfaction and expectations have not been thoroughly studied, despite their key role in the therapeutic strategy.

The relevance of quality of life for these patients is reflected in the fact that 65.9% consider an improvement in this aspect to be the main treatment goal. On the other hand, opinions among patients regarding treatment safety are divided and complete information is crucial to ensure treatment adherence. A recent study with GRAZAX^®^ [25] showed that all but one of the adverse reactions registered with the first dose occurred within the observation period specified in the SmPC for the used dose. The appropriate management of the patient´s reaction to this first dose, along with medical support, can help prevent treatment withdrawal. Physician-patient agreement, physician’s ability to deal with all the patient’s problems, and taking into consideration his/her expectations, feelings, and ideas are key for the final outcome.

It is generally accepted that better clinical benefits are achieved when treatment is started in early stages of a disease. In this study, patients who have had the disease for a shorter time, asses the effectiveness of the allergen immunotherapy tablet significantly better in comparison with patients who have suffered the condition for a longer time (<6 years: 78.2, 6–12 years: 76.4, >12 years: 69.7, *p* = 0.025).

In the Table 2, 33.9 and 36.5% feel that immunotherapy may be “sometimes” or “rarely” dangerous or may produce adverse events, respectively. With GRAZAX^®^ the adverse reactions are more frequent in the beginning of the treatment and, in many patients, with the administration of the first dose and most of reaction occurred during the observation period after the administration of the first dose. When the doctor explains the type of adverse reactions that can be expected by the treatment, and considering that GRAZAX^®^ is self-administered at patient´s home, the authors considered as relevant the perception of patients about the safety of treatment.

As for treatment adherence, most patients said to have received complete information about the disease and the treatment, although there was no written information (i.e., leaflets, etc.). Patients’ expectations are essential for adherence, and in this study a high percentage (between 25 and 40.5%) of the patients expected the treatment to have an effect “in days or weeks”. Patients should be encouraged to ask questions and be given clear verbal information by their physician, supplemented, whenever possible, by emotional support and written information packages.

Although it is clear that the intensity of the clinical management (number duration of the visits) affects various aspects of patient´s satisfaction, it is a non-linear effect, implying that other variables may influence this relationship. Determining whether physicians’ communication strategies have a direct effect on patient satisfaction ratings is not straightforward [26, 27]. Cohort studies to assess the effect of communication measures have to be designed and developed; however, strategies to promote treatment adherence should be based on the concept of user-friendly information and improved patient-health professional communication.

In summary, the level of satisfaction is closely related to the information given to the patients; satisfaction increases with the amount and type of explanations received from the physician (e.g., time explaining the disease, existing treatments, or specific information about the new tablet-based treatment). A better communication leads to increased patient knowledge, compliance, and satisfaction.
